# A case report of epileptic seizures caused by Rosai Dorfman disease followed by a literature review

**DOI:** 10.1097/MD.0000000000032553

**Published:** 2022-12-30

**Authors:** Zhanzhan Zhang, Aobo Zhang, Tao Zhang, Zongmao Zhao

**Affiliations:** a Department of Neurosurgery, The Second Hospital of Hebei Medical University, Shijiazhuang, China.

**Keywords:** case report, epilepsy, meningioma, Rosai Dorfman disease, sinus histiocytosis with massive lymphadenopathy

## Abstract

Rosai Dorfman disease is a rare benign histiocytoproliferative disorder that occurs in the intracranial area, which occurrs typically in lymph nodes. Extrapnodal Rosai Dorfman disease rarely develops in the central nervous system and is often a focal lesion based on the dura. Based on imaging and clinical symptoms, RDD may be misdiagnosed as meningioma, and some lesions can also occur in the brain parenchyma. In the case of benign disease, the final diagnosis is made by pathological tissue diagnosis. For chronic diseases, progression may be chronic or remitting and relapsing.

**Patient concerns:**

A 54-years-old man was hospitalized after experiencing paroxic convulsions and being unconsciousness. A head magnetic resonance imaging demonstrates a strip of lesions in the right parietal lobe. No obvious abnormality is found in the laboratory data.

**Diagnoses:**

We diagnosed meningioma of right parietal lobe and secondary epilepsy, and prescribed oral sodium valproate to treat him.

**Interventions:**

The lesion is located in the right parietal lobe on neuroimaging prior to surgery, which was taken for immunohistochemical examination.

**Outcomes:**

If it is found that immunohistochemistry reveals histiocytes are positive for CD68, S-100, but negative for CD1a, it is identified as RDD. For patients who are seizure-free following surgery, symptomatic management is used. Following parietal lesion resection, patients are seizure-free during the follow-up period (44 months).

**Lessons:**

Basing on studying and summarizing relevant literatures, RDD is described in the report in terms of its diagnosis, pathology, treatment, and clinical outcome, in order to improve the diagnosis and identification of intracranial RDD by physicians.

## 1. Introduction

Rosai Dorfman disease (RDD), also called Sinus histiocytosis with massive lymphadenopathy, is a rare, non-neoplastic benign histiocytosis of unknown cause. It was described as a specific clinicopathological entity and a benign and self-limiting condition by Rosai and Dorfman in 1969.^[1]^ In accordance with the extent of lesion, types of RDD can be classified as lymph node, cross-domain, and mixed. The most commonly involved site is lymph node. Typically, RDD arises in nodal sites, but 43% of RDD arises extra-nodally, involving the head and neck, paranasal sinuses, bone, respiratory tract, endocrine glands and skin, and the painless and bilateral cervical lymphadenopathy includes all characteristics of RDD. Systemic disease is characterized by fever, anemia, leukopenia, lymphocytopenia, enhanced ESR and polyclonal hypergammaglobulinemia (usually polyclonal). An estimated 5% of RDD is primary central nervous system without lymph node disease (5%). Central nervous system RDD, occurring in the brain or spine alonely, or simultaneously, is an isolated disorder that does not involve other systems.^[2]^ RDD remains controversial when it gets optimal treatment, based on the numerous case reports.

## 2. Clinical data

### 2.1. General information and clinical manifestations

A 54-years-old man was hospitalized after experiencing paroxic convulsions and being unconsciousness for 9 days. Past medical history includes history of hypertension and gastric bleeding. And there are no special personal history and similar family history. Physical examination: T 36.5ºC P 78/minutes R 19/minutes BP 140/96 mm Hg, no abnormalities were detected in the nerve examination, neck resistance was negative, and there is no apparent abnormality with the heart, lung, or abdomen; Muscle volume was normal in all limbs without any tremor in the muscle bundles. On the physical examination of the nervous system, the patient had normal tendon reflexes in his biceps, triceps, knees, and ankles, Babinski sign was negative on both sides, and Kernig sign was negative.

### 2.2. Routine examination and treatment

**Figure 1.**
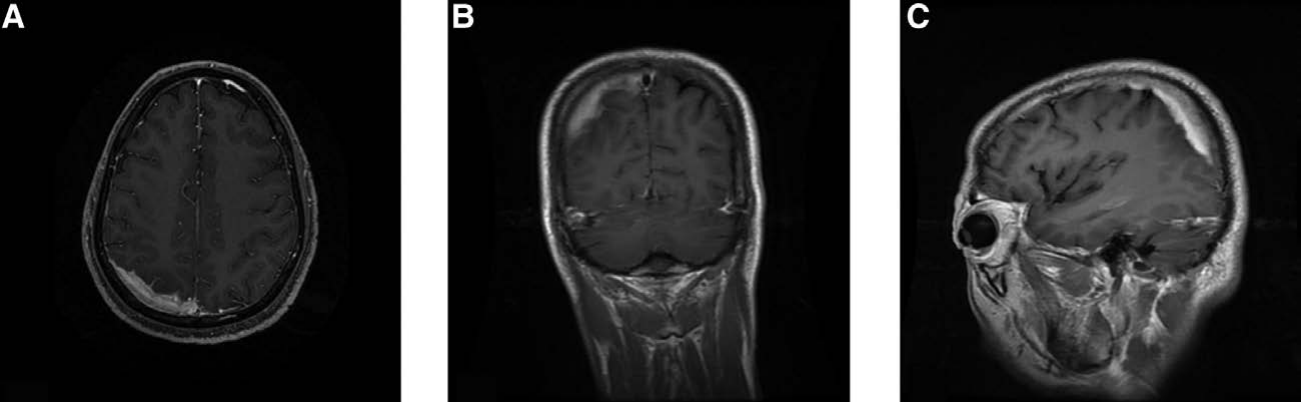
(A–C): Contrast-enhanced axial, coronal and sagittal MR image shows inhomogeneous enhancement of the right parietal lobe mass, involving the sagittal sinus.

A head magnetic resonance imaging (MRI) demonstrates: a strip of lesions in the right parietal lobe (Fig. 1A–C).

Laboratory data: Blood routine: Platelet distribution width 9.70 fL (normal range 7.40–11.00 fL); Coagulation routine: Fibrinogen 2.02g/L (normal range 2.38–4.98 g/L); Biochemical results: Indirect bilirubin 13.14 *μ*mol/L (normal range 1.70–10.20 *μ*mol/L); Alkaline phosphatase: 126.0 U/L (normal range 45.00–125.00 U/L), Total bile acid 14.8*μ*mol/L (normal range 0.00–10.00 *μ*mol/L), Creatinine 56.0 *μ*mol/L (normal range 57–97 *μ*mol/L), Carbon dioxide 29.8 mmol/L (normal range 22.00–29.00 *μ*mol/L); Human immunodeficiency virus negative.

### 2.3. Diagnosis and Treatment

**Figure 2.**
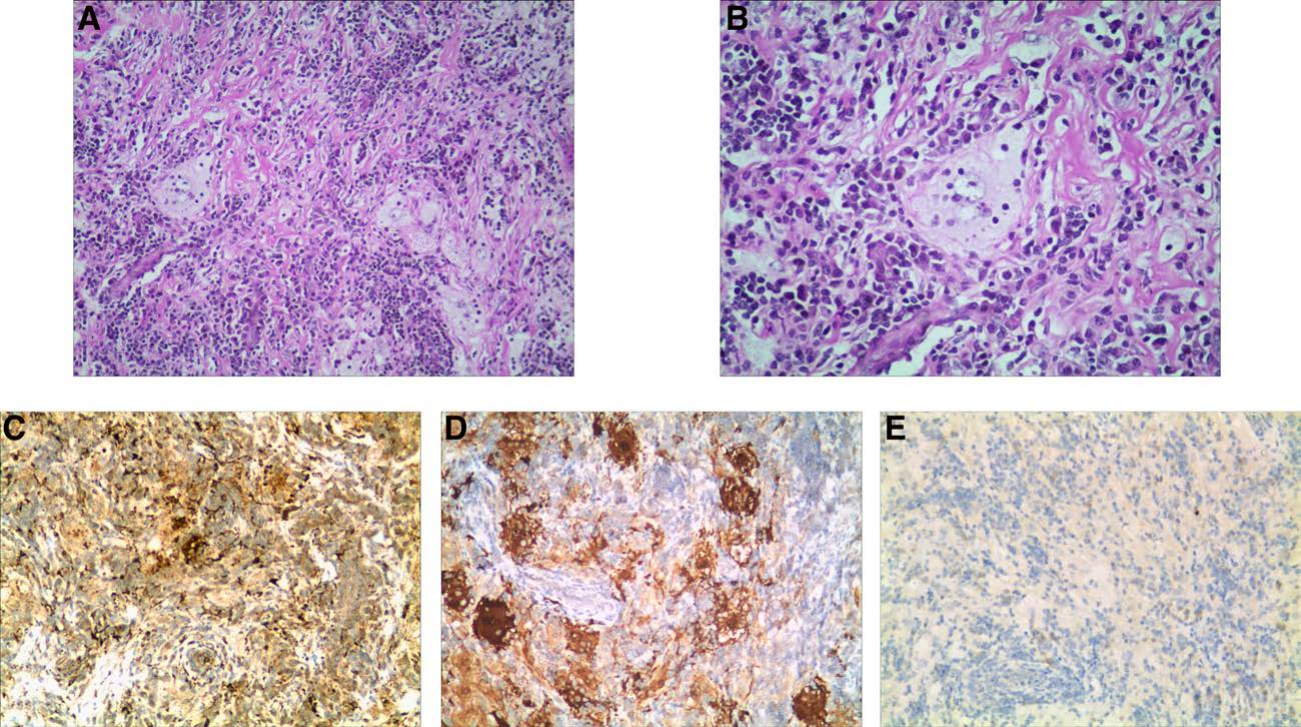
Pathological results (A): Lymphocytic infiltration, fibrous connective tissue hyperplasia (×100); (B): Tissue cells contain autologous lymphocytes (phagocytosis or “protrusion” of lymphocytes, ×200); (C–E): Immunohistochemical staining: positivity for CD68 and S-100, negativity for CD1a (x200).

Preoperative examination shows no abnormalities and undergoes surgical resection. During the operation, it is found that the tumor originates from the dura of the right parietal lobe that is located near the superior sagittal sinus, and the lamina dura is significantly thickened, with tumor size being about 5.5cm × 5.0 cm × 1.5 cm. Tumors are firmer in texture, have moderate blood supply, and sections of excised tumors are gray-yellow, with no clear border between tumor edge and normal tissue. The tumor and the involved dura are completely resected. The pathological diagnosis is RDD through the pathological phenotypes and immunophenotype. The immunohistochemistry shows: CD1a (-), CD20 (+), CD3 (+), CD34 (-), CD38 (+), CD68 (+), EMA (-), GFAP (-), Ki-67 (5%), Nestin (-), NeuN (-), Syn (-), Vimentin (+), S-100 (+) (Fig. 2A–E). The patient received postoperative treatment with antiepileptic drugs (Compound Sodium Valproate and Valproic Acid SR Tablets,500 mg/day, twice) for about 3 months. Further serum-related antibody screening and treatment were refused, so more detailed information could not be obtained, and the patient was cured and discharged smoothly after operation. During the follow-up 44-month, the patient was able to perform self-care activities and some degree of physical labor, and has now been seizure-free. During the follow-up period, because the patient recovered well after surgery and did not undergo cranial MRI examination, we cannot determine whether the intracranial lesions recurred.

## 3. Discussion

RDD, also called Sinus histiocytosis with massive lymphadenopathy, is a rare, non-neoplastic benign histiocytosis of unknown etiology, which is characterized by benignness and self-limitation in 1969.^[1]^ Some patients have a poor prognosis in published case reports, due to refractory, local invasion, or dissemination.

An unclear mechanism causes RDD, which might be caused by immune disorders triggered by specific infections. Scheel et al, reported skin RDD detected DNA of human herpetic virus 6 variant B, causing a biological reaction in histiocytes, or immune deficiency induced cell aggregation.^[3]^ Studies have reported that diseases such as hypothyroidism, autoimmune hemolytic anemia, human immunodeficiency virus and EB virus infection are complicated with RDD, suggesting that the relationship between RDD and immune dysfunction, that is immune-related RDD, may have a poor prognosis, which makes the verification of the hypothesis even more urgent.^[4–6]^ Studies have shown that mutations in KRAS and MAP2K1 and MAPK (ERK) pathway activation are detected frequently in RDD specimens, with mutations in KRAS and MAP2K1 being mutually exclusive in about 1-third of Rosai Dorfman patients.^[7]^ Next generation sequencing reveals that the BRAF gene exhibites a single somatic mutation in exon 12, and cell proliferation in most central nervous system tumors is associated with BRAF V600E mutations, suggesting that RDD may be a tumor lesion.^[6,8,9]^ Therefore, we speculate that BRAF-mutant patients with targeted therapy for RDD improve their survival significantly by using inhibitors of BRAF in the future. It has also been reported that a mutation in the nucleoside transporter gene SLC29A3 is the molecular basis of familial RDD and has a certain genetic predisposition.^[10]^

Clinically, the bilateral cervical lymphadenopathy, which causes no pain, is the most common type. Symptoms of systemic disease include fever, anemia, leukocytosis, lymphocytopenia, elevated erythrocyte sedimentation rate, and polyclonal hypergammaglobulinemia (usually polyclonal), but none of the above symptoms are specific. When some patients are diagnosed as RDD, they have already suffered from auto-immunodeficiency diseases such as rheumatoid arthritis and systemic lupus erythematosus.^[11]^ In this case, patients admit that they are experiencing paroxic convulsions and unconsciousness. Laboratory data are basically normal, and further examination is required to diagnose RDD.

According to the literatures, approximately 75% of central nervous system (CNS) RDD is intracranial and rarely involves the spine, approximately twice the incidence of spine, and more rarely involves the brain and spinal cord. Intracranial lesions often occur in suprasellar region, convexity, parasagittal area, cavernous sinus, petroclival region, and the dura mater and brain parenchymal tissue are involved individually or simultaneously. Recent statistics show that: depending on the location of the tumor, clinical symptoms are headache (45.1%), focal neurologic dysfunction (32.6%), visual symptoms (32.1%), epilepsy (24.6%), cerebral neuropathy (11.6%), cerebellar dysfunction (11.2%), and cognitive decline (5.8%), and spinal lesions may follow with hemiplegia or paraplegia.^[12]^ In this case, epileptic seizures are the main symptoms, and it is found that there are intracranial lesions through imaging examination.

As seen on imaging, CNS RDD usually appear as multiple or solitary dura-based masses, while CT scans are characterized by homogeneous, lobulated isodense or hyperdense mass to brain tissue, which may cause erosion of the skull. MRI usually shows that T1WI represents isointence in tumor, while T2WI represents hypointense or isointence in tumor, and an uneven or uniform enhancement and a clear boundary are visible after contrast-enhanced scanning. There is an obvious dural tail sign on enhanced MRI in almost all cases, which is easily diagnosed as meningioma.^[2]^ Neuroimaging in some patients shows that the intracranial lesion is unsurrounded by edema. Some scholars believe that RDD may follow with inflammation rather than tumor on neuroimaging, which has been suspected to be caused by infections, particularly virus infections, such as inflammatory pseudo-tumor, sarcoidosis, tuberculosis and Langerhans cell histiocytosis.^[2]^

In order to make a definite diagnosis of RDD, a histopathologic and immunohistochemical examination is required, which is characterized by phagocytosis or “protrusion” of lymphocytes. The condition exhibits mature lymphocytes, plasma cells and neutrophils with fiber hyperplasia, and tissue cells often contain autologous lymphocytes as a diagnostic basis.^[1,2]^ CNS RDD lesions present as gray or gray-brown, or yellow-brown masses with an intracranial location attaching to the dura mater. There is a positive immunohistochemistry for the S-100 protein and CD68 marker in RDD, and a negative immunohistochemistry for CD1a, which should be distinguished from lymphoplasmacytic meningioma, plasma cell granuloma, Langerhans cell histiocytosis and Hodgkin lymphoma.

It is possible to evaluate treatment protocols of RDD in different ranges of lesion sizes and locations for the adjacent brain tissue, clinical manifestations. Tumor biopsies may have some risk, while some tumor patients maynot be inoperable, such as the cavernous sinus invasion. This case is diagnosised as a meningioma, and surgical resection is the first therapy to relieve neurological symptoms and make a pathological diagnosis. Reoperation goes well for the patient, and epileptic seizures completely disappear.^[4]^ For some patients with subtotal resection, adjuvant therapy, such as radiotherapy, chemotherapy and steroid hormones, is alone or in combination to reduce postoperative recurrences. There is also no clear explanation of the mechanism and the efficacy shows individual differences, so this disease cannot be treated according to a standard protocol.

Due to the rare cases of intracranial RDD, clinicians have insufficient understanding and limited experience cannot guide clinical therapy. If multicenter collaborative works are recommended, the exact molecular mechanisms would be clarified from the molecular research level. Therefore, the best way to avoid overtreating is to make long-term clinical studies and follow-up summaries.

## Author contributions

**Conceptualization:** Zhanzhan Zhang, Zongmao Zhao.

**Data curation:** Aobo Zhang.

**Formal analysis:** Zhanzhan Zhang, Tao Zhang, Zongmao Zhao.

**Funding acquisition:** Zongmao Zhao.

**Project administration:** Zongmao Zhao.

**Resources:** Tao Zhang.

**Supervision:** Zongmao Zhao.

**Validation:** Zongmao Zhao.

**Writing – original draft:** Zhanzhan Zhang.

**Writing – review & editing:** Zongmao Zhao.
